# A critical review of citrulline malate supplementation and exercise performance

**DOI:** 10.1007/s00421-021-04774-6

**Published:** 2021-08-21

**Authors:** Lewis A. Gough, S. Andy Sparks, Lars R. McNaughton, Matthew F. Higgins, Josh W. Newbury, Eric Trexler, Mark A. Faghy, Craig A. Bridge

**Affiliations:** 1 Research Centre for Life and Sport Science (CLaSS), Human Performance and Health Research Group, Birmingham City University, Birmingham, UK; 2 Sports Nutrition and Performance Research Group, Department of Sport and Physical Activity, Edge Hill University, Ormskirk, UK; 3 University of Derby, Derby, UK; 4 Stronger By Science LLC, Raleigh, NC USA; 5 Human Sciences Research Centre, University of Derby, Derby, UK

**Keywords:** Metabolism, High-intensity exercise, Supplements, Resistance training, Nitric oxide

## Abstract

As a nitric oxide (NO) enhancer, citrulline malate (CM) has recently been touted as a potential ergogenic aid to both resistance and high-intensity exercise performance, as well as the recovery of muscular performance. The mechanism has been associated with enhanced blood flow to active musculature, however, it might be more far-reaching as either ammonia homeostasis could be improved, or ATP production could be increased via greater availability of malate. Moreover, CM might improve muscle recovery via increased nutrient delivery and/or removal of waste products. To date, a single acute 8 g dose of CM on either resistance exercise performance or cycling has been the most common approach, which has produced equivocal results. This makes the effectiveness of CM to improve exercise performance difficult to determine. Reasons for the disparity in conclusions seem to be due to methodological discrepancies such as the testing protocols and the associated test–retest reliability, dosing strategy (i.e., amount and timing), and the recent discovery of quality control issues with some manufacturers stated (i.e., citrulline:malate ratios). Further exploration of the optimal dose is therefore required including quantification of the bioavailability of NO, citrulline, and malate following ingestion of a range of CM doses. Similarly, further well-controlled studies using highly repeatable exercise protocols with a large aerobic component are required to assess the mechanisms associated with this supplement appropriately. Until such studies are completed, the efficacy of CM supplementation to improve exercise performance remains ambiguous.

## Introduction

Considerable research attention has recently been placed on the physiological signalling molecule, nitric oxide (NO) (Jones et al. 2020). Augmenting NO synthesis through exogenous substances may improve skeletal muscle function and performance through improved blood flow, contractility, and mitochondrial respiration (Stamler and Meissner 2001). Typical strategies to increase NO activity include the ingestion of green leafy vegetables and/or beetroot juice and L-citrulline (Jones 2020). Indeed, L-citrulline is known to exert positive effects on exercise performance and recovery (Gonzalez and Trexler 2020). More recently, however, a direct NO precursor called citrulline malate (CM) has been touted to have ergogenic potential, which is the combination of L-citrulline and malate (Gonzalez and Trexler 2020). The mechanisms of CM might be more far-reaching as a result, due to the synergistic impact of both components (i.e., L-citrulline and malate) at the intramuscular level (Wax et al. 2015). Specifically, malate has been suggested to increase the rate of ATP production by mitigating lactate production during states of high flux; and by doing so allowing for continued pyruvate and energy production (Wax et al. 2016). Furthermore, the malate-aspartate shuttle (MAS) may be more efficient following CM ingestion, thereby improving ATP availability (Wu et al. 2007; Agudelo et al. 2019). Based on these promising findings and additional mechanisms compared to L-citrulline supplementation alone, it is plausible to suggest CM supplementation could be a worthwhile ergogenic aid.

Since the early work of Bendahan et al. (2002), research has primarily focused on the potential ergogenic effects of CM supplementation on resistance exercise performance. In an early study, a greater contribution of oxidative ATP synthesis (34% increase) to energy production was observed with chronic ingestion of CM for 15 days (6 g^.^day) (Brendahan et al. 2002). This study, however, focused on sedentary individuals who complained of fatigue and included no placebo condition and therefore, the application to athletes is limited. Nonetheless, recent work has addressed these limitations and investigated acute doses of CM against a placebo and the associated effects on short-term exercise that encompasses a large anaerobic component in trained and untrained individuals. To date, equivocal responses to CM supplementation have been reported, which makes the performance-enhancing potential ambiguous. Moreover, CM ingestion could improve acute recovery from exercise due to the augmentation of blood flow and the indirect increase in nutrient delivery and clearance of waste metabolites (Wax et al. 2015; Glenn et al. 2017). This could have important implications for athletes who may have minimal recovery from competition/training due to the high frequency of exercise bouts in their schedules (e.g., team sports, track and field). This review therefore builds upon a previous review by Gonzalez and Trexler (2020) who recently discussed the efficacy CM ingestion to improve exercise performance within a larger, more general review of NO enhancing supplements, by offering a more in-depth discussion on the potential mechanisms associated with CM supplementation. This is followed by a discussion of findings to date in respect of improving exercise performance and/or recovery, including modifying factors such as the exercise type and duration, ingestion strategy (dose and timing), and the safety of CM supplementation.

## Proposed ergogenic mechanisms

The ingestion of CM was originally prescribed to enhance the muscle performance of patients suffering from asthenia and to facilitate the recovery of muscle function resulting from acute diseases (Brendahan et al. 2002). As an organic salt, CM and is formed through the combination of L-citrulline (C_6_H_13_N_3_O_3_), a non-essential amino acid involved in the urea cycle, and malate (or malic acid, C_4_H_6_O_5_), a tricarboxylic acid (TCA) intermediate (Bendahan et al. 2002; da Silva et al. 2017; Glenn et al. 2017; Wax et al. 2015). Outside of the common practice of oral supplementation of beetroot juice, L-citrulline ingestion has been the most researched nutritional strategy to stimulate NO production (Gonzalez and Trexler, 2020). This is likely explained by studies that show L-citrulline ingestion to be the most efficient means of elevating plasma arginine concentrations, which in turn, produces NO (Schwedhelm et al. 2008).

**Fig. 1:**
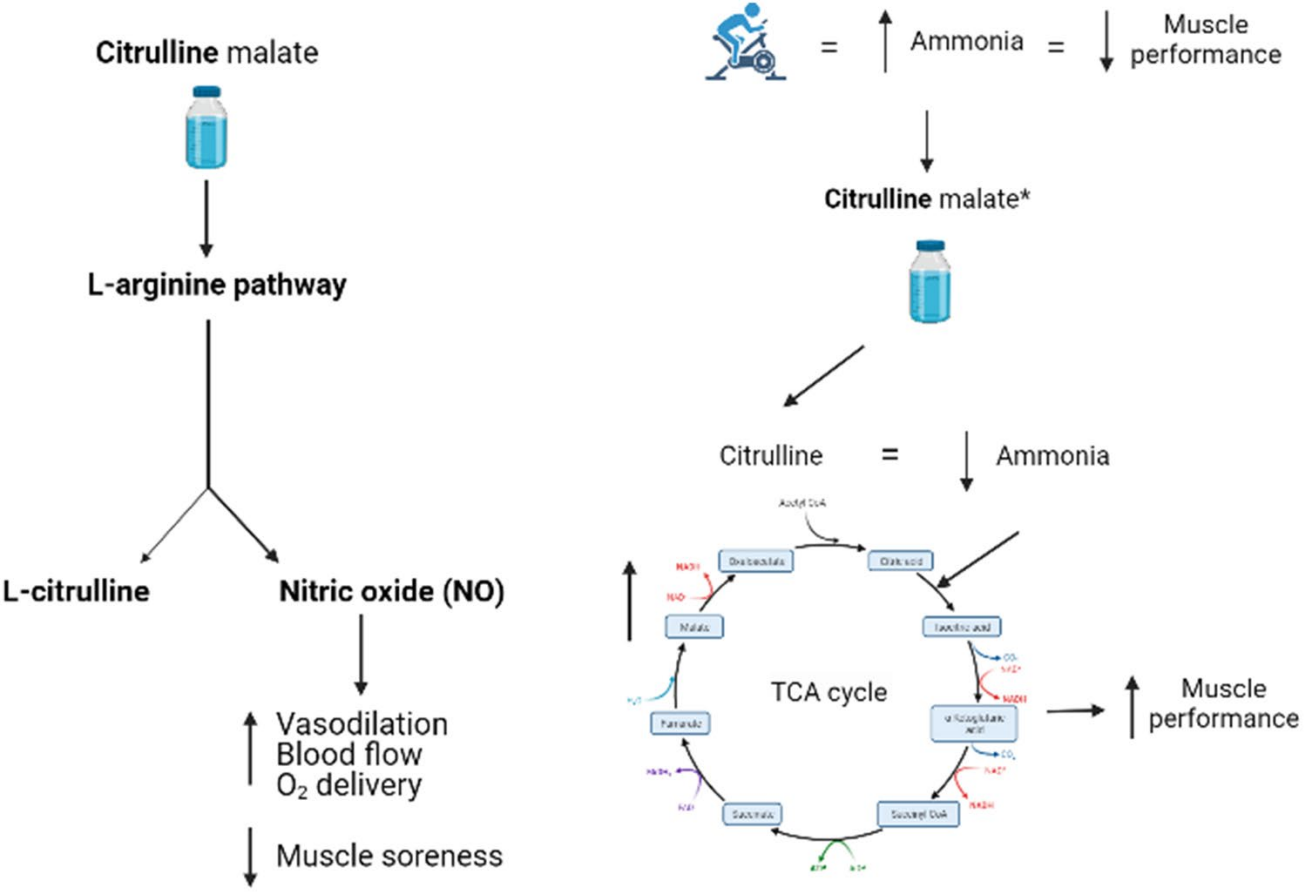
A schematic representation of the mechanisms associated with citrulline malate supplementation. Bold denotes the active ingredient. Left: NO-derived mechanism, Right: Ammonia clearance mechanism. *Denotes evidence is either speculative or has only been observed in mice. (Schematic created in BioRender.com)

The proposed mechanism for CM ingestion is firstly dependent on the citrulline component via the L-arginine-NO pathway, such that following NO synthesis the smooth muscle may relax leading to vasodilation (Vanhoutte et al. 2016; Fig. 1). In turn, these vasodilatory properties may improve the delivery of blood (and oxygen) to and from the active musculature during exercise (Wax et al. 2015). Trexler and colleagues (2019a, 2019b) have recently questioned this mechanism, however, using the near-infrared spectroscopy (NIRS) technique to quantify blood flow. Indeed, Trexler et al. (2019a) reported 8 g CM ingestion 2 h prior to exercise had no effect on muscle blood flow (CM: 3.78 ± 0.26, placebo: 3.72 ± 0.26 ml.min^−1^.100 ml^−1^) or oxygen consumption (CM: 1.15 ± 0.11, placebo: 1.16 ± 0.11 mLO_2_.min^−1^.100 g^−1^) during 3 min of leg extension exercise (one rep every 4 s). These findings were replicated during maximal effort leg extension exercise consisting of 5 sets of 30 maximal-effort concentric leg extensions interspersed with 1 min of passive rest between sets (Trexler et al. 2019b). The authors findings were likely due to the small rises in NO observed in these studies, as they were not significantly different to the placebo (CM: 15.3 ± 1.1; placebo: 13.4 ± 1.1 μmol·L; Trexler et al. 2019b). Alternatively, the failure to select a dynamic whole-body exercise might have led to no improvement, as this might not have sufficiently stressed the aerobic mechanisms associate with ingestion of this supplement. Finally, whilst the use of NIRS is widely considered a reliable technique (Lucero et al. 2018), it is unclear if this technique is sensitive enough to detect changes following supplement intake. The evidence to date specifically on CM supplementation suggests that enhanced blood flow caused by the citrulline component is not the acting mechanism, although further experimental research is required.

An alternative mechanism could be from the citrulline component of CM, as this may assist with ammonia elimination during the urea cycle (Bendahan et al. 2002; Fig. 1). This is important due to the increased ammonia production observed during high-intensity exercise, and the association of these changes with muscle fatigue (Gonzalez and Trexler 2020). Specifically, high ammonia concentrations facilitate the production of lactate during anaerobic glycolysis by activating phosphofructokinase (PFK). In turn, this prevents oxidative metabolism of pyruvate to acetyl-CoA and hinders the ATP supply to skeletal muscle (Hargreaves and Spriet 2020). Citrulline may exert its ergogenic effects through this mechanism by detoxifying ammonia during high-intensity exercise, thereby enhancing the aerobic utilization of pyruvate and ATP supply to skeletal muscle (Gonazalez and Trexler, 2020). Indeed, Takeda et al. (2011) reported ingestion of citrulline (250 mg.kg^−1^ BM) reduced ammonia accumulation by 90.1 µg/dL^−1^ (citrulline: 351.3 ± 35.3 vs. control: 441.4 ± 61.3 µg.dL^−1^) and increased time to exhaustion swimming by approximately 9 min in mice (citrulline = 24 min, placebo = 15 min). Importantly, post-exercise lactate concentrations were lower for the citrulline-supplemented group, which in turn, supports the mechanism of enhancing aerobic utilization during exercise. This has not been replicated in humans, however, and as such, it remains to be confirmed if this mechanism occurs during human exercise.

**Fig. 2:**
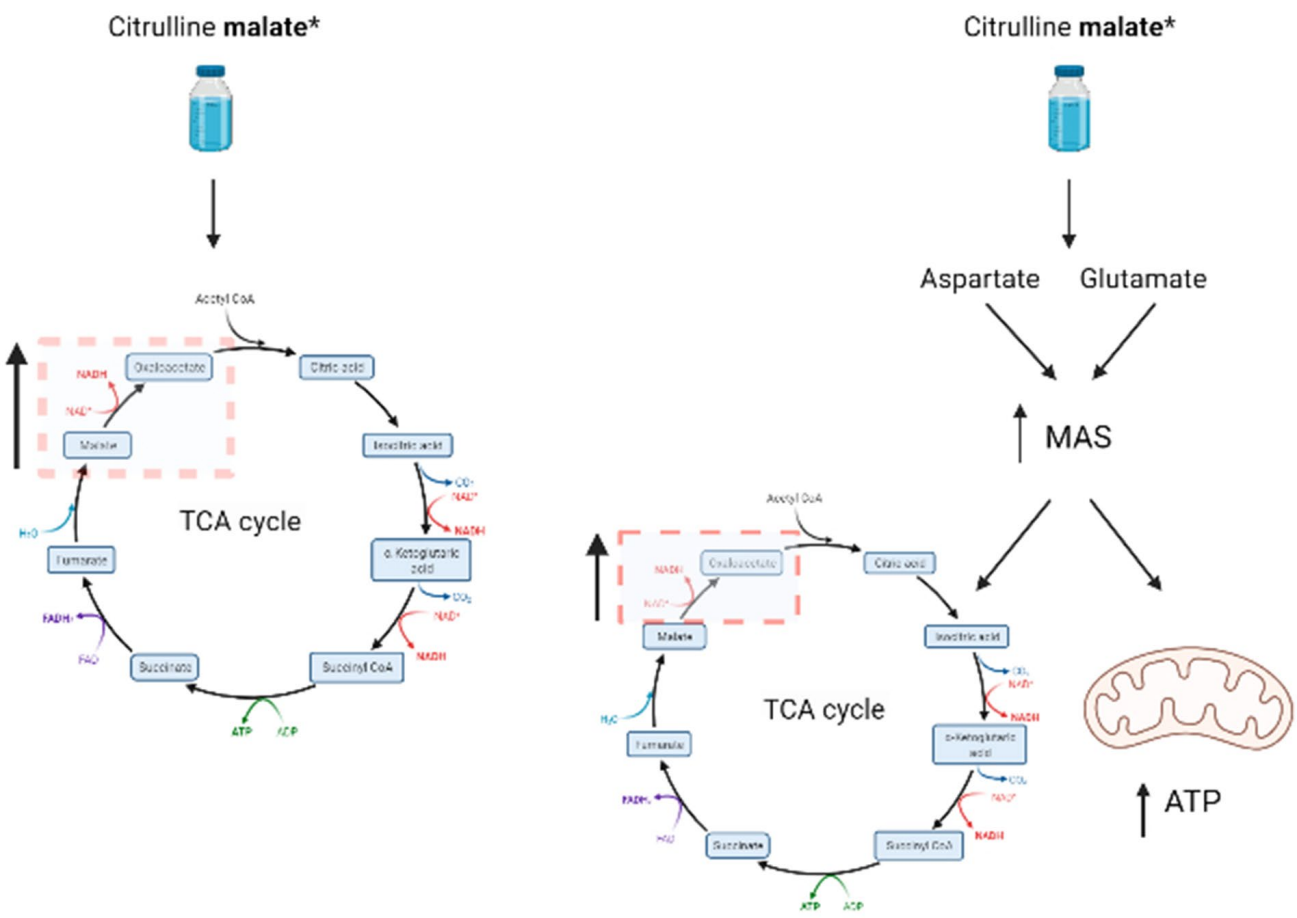
A schematic representation of the mechanisms associated with citrulline malate supplementation. Bold denotes the active ingredient. Left: Increased bioavailability of malate mechanism, Right: Increased efficiency of Malate Aspartate Shuttle (MAS). *Denotes evidence is either speculative or has only been observed in mice. (Schematic created in BioRender.com)

Greater ergogenic benefits may be possible in addition to the L-citrulline mechanisms with the addition of malate (Fig. 2). The primary role of malate is to function as a tricarboxylic acid cycle intermediate, which may play an important role in the rate of ATP production (Brendahan et al. 2002). One of the most critical controls of the rate of aerobic ATP production is oxaloacetate, and as malate is dehydrogenated into this compound in the TCA cycle, it may offer an explanation to the purported additive effects of CM over L-citrulline alone (Brendahan et al. 2002). Equally, malate is suggested to play a critical role (amongst four other TCA intermediates) in ancillary reactions that can alter the concentrations of TCA intermediaries and result in positively affecting the fluxes in and out of the TCA cycle (Gibala et al. 2000). Whilst these mechanisms sound promising, no study to date involving CM supplementation and exercise performance has provided evidence of these mechanisms occurring. Admittedly, this is difficult given the complexities and lack of measurement techniques, and most evidence supporting this theory to date is based on mathematical approaches/modelling. It is not possible therefore to confirm at this time whether they are important during exercise. Nonetheless, the synergistic mechanisms of L-citrulline and malate (i.e., CM) do offer hope that these mechanisms could synergistically improve exercise performance. Future research should directly compare L-citrulline vs. CM ingestion to assess if the addition of malate does offer additive benefits to exercise performance.

A contemporary mechanism may be either through the increase in gene expression, or increased efficiency of the MAS, which is caused by elevations in PGC-1α in exercising musculature. These physiological changes can elevate aspartate and glutamate levels and increase the expression of glycolysis and MAS genes (Wu et al. 2007; Agudelo et al. 2019). As a result, increases in the transfer of fuel-derived electrons to mitochondrial respiration may occur, which in theory, should improve energy utilisation (Wu et al. 2007; Fig. 2). Indeed, evidence in mice has shown that supplementation of L-malate increased the activity of malate dehydrogenase (MDH) in a dose–response manner, as higher doses reported more activity (Control mice: 15.7 ± 2.4 vs. 0.2 g.kg^−1^ BM^.^day^−1^ dose: 24.3 ± 3.5 vs. 0.6 g.kg^−1^ BM^.^day^−1^ dose: 26.4 ± 3.7) (Wu et al. 2007). Mechanistically, as MDH is a rate-limiting enzyme for MAS, an increase in activity could suggest an increase in efficiency and therefore ATP supply. Similarly, MDH yields oxaloacetate by dehydrogenating L-malate to increase overall rates of TCA flux and MAS. Support for these mechanisms can be found in Wu et al. (2007), such that time to exhaustion swimming was improved in both the 0.2 g.kg^−1^ BM^.^day^−1^ g (+ 26.1%; 620 ± 141 s) group and 0.6 g.kg^−1^ BM^.^day^−1^ (+ 28.5%; 631 ± 134 s) versus the control (491 ± 145 s). However, no data is available from using human participants to corroborate these findings in mice, whilst Wu et al. (2007) also chronically supplemented L-malate, not CM. Further research in humans is therefore required to confirm this mechanism.

Supplementation of CM is suggested to reduce muscle soreness from exercise, via the purported increased blood flow mechanism (Wax et al. 2015; da Silva et al. 2017). This could be important for either subsequent performance when recovery between bouts is limited (i.e., track and field) and/or overall quality and quantity of chronic training. In a large study of forty-one men, ingestion of 8 g CM 1 h before a resistance training bout led to decreases in muscle soreness at both 24 h (-40%) and 48 h (-41%), respectively, compared to a placebo (Perez-Guisado et al. 2010). However, there was no subsequent performance bout to substantiate whether the reduction in soreness translates to improved performance. In a more thorough study (da Silva et al. 2017), CM supplementation (6 g, 1 h before exercise) failed to improve muscle soreness when more reporting points (24, 48, and 72 h) were used compared to Perez-Guisado et al. (2010). Importantly, da Silva et al. (2017) also reported no improvement in subsequent performance for neither leg press exercise, nor hack squat to exhaustion. Whilst the lack of differences observed could be attributed to the low 6 g dose adopted by da Silva et al. (2017), a more recent study has corroborated these findings using an 8 g dose, 1 h before exercise (Chappel et al. 2018). Based on the current evidence that CM supplementation does not enhance blood flow and thereby increase nutrient delivery and/or remove waste metabolites, this might explain why no positive impact has been reported to date for muscle soreness and subsequent exercise performance.

## Dose, timing, and safety

The most commonly employed dose of CM is a single acute 8 g dose (Gonzalez and Trexler 2020), which appears to reflect early work observing performance benefits during resistance exercise using this dose (Perez-Guisado et al. 2010; Table 1). However, an earlier dose–response study (Moinard et al. 2008) investigated the pharmacokinetics of 2, 5, 10, and 15 g of citrulline within eight healthy volunteers and demonstrated that larger doses could be more appropriate. The authors reported that peak citrulline concentration occurred with a 15 g dose (3849 ± 190 μmol.l^−1^), which was significantly higher (+ 28.4%) compared to 10 g citrulline (2756 ± 170 μmol.l^−1^). It is plausible such larger doses (i.e., > 10 g) will increase the likelihood of securing an ergogenic benefit during exercise, which questions the use of a 8 g dose in most research to date. It is worth noting, however, that Moinard et al. (2008) only used citrulline, rather than the combined intake of CM. The extent to which the malate may influence the peak concentration and pharmacokinetics is therefore currently unknown and warrants further investigation. Indeed, to achieve an intake of 10 g of L-citrulline, ingestion of around 3–5 kg of fresh watermelon (highest concentration of all foods) would be required and this is not only impractical for athletes (Davis et al. 2011), but would also not contain a sufficient dose of the malate component. Given the large dose required for any possible ergogenic effect, supplementation is only practical via ingestion of marketed sports supplements or the raw chemical compound.

**Table 1:** Ergogenic influences of citrulline malate on resistance exercise performance

Study	Participants	Dose (C-M ratio) and Timing	Experimental design	Functional Measure/test	Resistance Exercise Protocol*	Resistance Performance Outcomes	Other measures
Bendahan et al. (2002)	Sedentary males symptomatic of fatigue (*n* = 18) Age: 31 ± 9 years	CM: 2 g ingested 3 × per-day (6 g) for 15 days	Single group treatment for 15 days	Dynamic finger flexions using slide weight and displacement transducer – power (w) ^31^P magnetic resonance spectroscopy	Finger flexions performed at 1.5 s intervals lifting a 6 kg weight for 3 min Performed: 2 × before ingestion CM 3 × during ingestion CM 1 × after ingestion CM	Power (w) ↑ during CM ingestion CM when compared with before ingestion (2.0 + 0.1 vs. 1.7 ± 0.1)	Delta change in pH per unit of power ↓ during CM ingestion compared with before ingestion (0.24 ± 0.02 vs. 0.29 ± 0.03) Rate of oxidative ATP production (%EC) ↑ during CM ingestion (74 ± 4 vs. 54 ± 12) Rate of PCr resynthesis (mmol/min) ↑ during CM ingestion (24.2 ± 3.2 vs. 16.9 ± 2.9)
Perez-Guisado et al. (2010)	Resistance trained males (n = 41) Age: 30 ± 8 years	CM: 8 g PLA: 10 g sugar and 60 mg sodium saccharine Single dose 1-h prior	Randomized, double-blind, cross-over design 7-day wash out	Dynamic concentric and eccentric muscular endurance/strength using barbell	4 sets at 80% 1RM until failure before and then again after a pectoral training workout 1-min rest between sets Barbell bench press	Bench press: total reps ↑ in CM vs. PLA before workout: Set 3 (8.2 ± 1.6 vs.7.4 ± 1.6) Set 4 (7.1 ± 1.7 vs. 6.0 ± 1.6) Total reps ↑ in CM vs. PLA after workout: Set 1 (10.3 ± 1.8 vs. 9.2 ± 2.1) Set 2 (8.4 ± 1.8 vs. 6.9 ± 2.0) Set 3 (6.9 ± 1.7 vs. 5.1 ± 1.8) Set 4 (5.5 ± 1.5 vs. 3.6 ± 1.4)	Muscle soreness following 24-h ↓ 39.7% in CM vs. PLA 48-h ↓41.8% in CM vs. PLA
Wax et al. (2015)	Resistance trained males (n = 12) Age: 22 ± 1 years	CM: 8 g PLA: Maltodextrin and aspartame Single dose 1-h prior	Randomized, double-blind, counter-balanced, cross-over design 7-day wash out	Dynamic concentric and eccentric muscular endurance using machines	5 sets at 60% 1RM until failure 3-min rest between sets Leg press Hack squat Leg extension	Leg press: total reps ↑ in set 5 in CM vs. PLA Hack squat: total reps ↑ in sets 4 and 5 in CM vs. PLA Leg extension: total reps ↑ in set 5 in CM vs. PLA (mean data not reported)	Blood lactate ↔ HR ↔ Blood pressure: Systolic ↔ Diastolic ↔
Wax et al. (2016)	Resistance trained males (n = 14) Age: 23 ± 2 years	CM: 8 g PLA: Maltodextrin and aspartame Single dose 1-h prior	Randomized, double-blind, counter-balanced, cross-over design 7-day wash out	Dynamic concentric and eccentric muscular endurance using body weight exercises	3 sets until failure 3-min rest between sets Chin-up Reverse chin-up Push-up	Chin-up: total reps: ↑ in CM vs. PLA (32.2 ± 5.6 vs. 28.4 ± 7.1) Reverse chin-up total reps ↑ in CM vs. PLA (32.1 ± 7.1 vs. 26.6 ± 5.6) Push-up total reps: ↑ in CM vs. PLA (97.7 ± 36.1 vs. 89.1 ± 37.4)	Blood lactate: ↔ HR: ↔ Blood pressure Systolic: ↔ Diastolic: ↓
Glenn et al. (2017)	Resistance trained females (n = 15) Age 23 ± 3 years	CM: 8 g + 8 g dextrose PLA: 8 g dextrose Single dose 1-h prior	Randomized, double-blind, cross-over design 7-day wash out	Dynamic concentric and eccentric muscular endurance/strength using barbell and plate loaded leg press	6 sets at 80% 1RM until failure 1-min rest between sets Bench press Leg press	Bench press: total reps ↑ in CM vs. PLA (34.1 + 5.7 vs. 32.9 + 6.0) Leg press: total reps ↑ in CM vs. PLA (66.7 ± 30.5 vs. 55.1 ± 20.6)	Bench press RPE: ↓ Leg press RPE: ↔ Bench press HR: ↔ Leg Press HR: ↔
Gonzalez et al. (2018)	Recreational resistance trained males (n = 12) Age: 21 ± 2 years	CM: 8 g PLA: Flavored water Single dose 40-min prior	Randomized, double-blind, placebo-controlled, counter-balanced, cross-over design ~ 7-day wash out	Dynamic concentric and eccentric muscle endurance/Hypertrophy using barbell Dynamic Power measured with linear position transducer	5 sets × 15 reps at 75% 1RM 2-min rest between sets Barbell bench press	Total reps: ↔ Peak power: ↔ Mean power: ↔ Fatigue index: ↔	RPE: ↔ Muscle thickness (cm): ↔ Subjective feelings of focus, energy, fatigue, and muscle pump: ↔
Chappell et. al. (2018a)	Recreational resistance trained males (n = 11) and females (n = 4) Age: 24 ± 2 years	CM: 8 g (1.11: 1) PLA: 6 g Citric acid Single dose 1-h prior	Randomized, double-blind, placebo-controlled, counter-balanced, cross-over design 7-day wash out	Isokinetic-dynamometer: Isometric force max Concentric force max Eccentric force max	10 sets × 10 reps 70% of Concentric force max 1-min rest between sets Leg curl – knee extensor and flexor strength	Total Reps: ↔ Isometric force max: ↔ Concentric force max: ↔ Eccentric force max: ↔	Blood lactate: ↔ Quadriceps muscle soreness: ↑ 25.7%, 41.1%, 37.3% in CM vs. PLA at 24, 48 and 72 h following exercise, respectively
Chappell et al. (2018b)	Recreational resistance trained males (n = 12) and females (n = 17) Age: 26 ± 8 years	CM: 8 g (1.11: 1) PLA: 6 g Citric acid Single dose 1-h prior	Randomized, double-blind, placebo-controlled, counter-balanced, cross-over design 7-day wash out	Dynamic concentric and eccentric muscular endurance/strength using barbell	10 sets × 10 reps at 80% 1RM 1-min rest between sets Barbell Curls	Total reps: ↔	Blood lactate: ↔ Creatine Kinase: ↔ Upper and lower arm muscle soreness: ↓ in CM vs. PLA at 24, 48 and 72 h (individual data not reported)
Farney et al. (2019)	Recreationally trained males (n = 6) and females (n = 6) Age: 24 ± 4 years	CM: 8 g PLA: 20 oz sugar free water CON: No drink Single dose 1-h prior	Single-blind, balanced, randomized, cross-over design 7-day wash out	Isokinetic-dynamometer Power Fatigue Index	1 set × 15 reps at 180^o^sec Leg extension	Measured following high intensity exercise body weight session: Total Reps: ↔ Peak torque: ↔ Peak power: ↔ Fatigue index: ↔	Lactate: ↔ Heart rate: ↔
da Silva et al. (2017)	Recreational active males (n = 9) Age: 24 ± 3 years	CM: 6 g PLA: Corn starch Single dose 1-h prior	Double-blind, randomized, crossover design 7-day wash out	Recovery of Dynamic muscular endurance	1 set at 100% of 10 RM Machine Leg press Machine Hack Squat	Measured into 24, 48, 72 h recovery only following resistance exercise 3 sets at 90% of 10RM (2 min rest): Total no. Reps: ↔	RPE: ↔ Lactate: ↔ Creatine Kinase: ↔ Muscle soreness: ↔ Lactate: ↔ Testosterone-cortisol: ↔ Electromyography: ↔
Trexler et al. (2019a)	Recreationally active men (n = 27) Age: 22 ± 4 years	8 g dose 2-h prior to exercise	Double-blind, randomized placebo-controlled design	Maximal concentric leg extensions	5 sets × 30 reps	No performance enhancing effect: ↔ versus placebo	NO_x_ pre or post-exercise**:** ↔ vs. PLA Blood flow: ↔ vs. PLA Metabolic efficiency: ↔ vs. PLA Hormonal response: ↔ vs. PLA
Trexler et al. (2019b)	Recreationally active men (n = 27) Age: 22 ± 4 years	8 g dose 2-h prior to exercise	Double-blind, randomized placebo-controlled design	Submaximal isotonic leg extensions	25% of maximal voluntary contraction torque	No performance measure	Muscle blood flow: ↔ Oxygen consumption: ↔ Respiratory exchange ratio: ↔ Indirect calorimetry: ↔
Hwang et al. (2018a, b)	Resistance trained males (n = 75) Age: 21 ± 2 years	CM = 2 g per day for 8 weeks L-citrulline & glutathione (LG): 200 mg^.^day of GSH + 2 g^.^day of L-citrulline PLA: 2.52 g^.^day of cellulose	Double-blind, randomized placebo-controlled design	Maximal muscular Strength (1RM test) adaptation evaluated over 8 weeks	1 RM testing: Free weight bench press Angled leg press Performed at baseline, 4 and 8 weeks training	Bench press Baseline: ↔ 4 weeks: ↔ 8 weeks: ↔ Angled leg press Baseline: ↔ 4 weeks: ↔ 8 weeks: ↔	Body mass: ↔ Fat mass: ↔ Body water: ↔ Lean Mass: ↑ at week 4, but not week 8 in LG vs. PLA

Citrulline malate (CM); placebo (PLA), control (CON), L-citrulline & glutathione (LG); *Reps* repetitions; *RM* repetition max; *RPE* ratings of perceived exertion; *HR* heart rate; *NO*_*X*_ plasma nitrate; ↑ significant increase; ↔ no significant change; ↓ significant decrease

*Reps represent the target reps for the participants

There appears to be limited diversity in the timing of CM ingestion with most studies opting for 1-h ingestion before exercise (Gonzalez and Trexler 2020). This approach seems to be informed by previous research reporting ergogenic effects using similar dosing strategies (Wax et al. 2015, 2016; Farney et al. 2019), rather than the time to reach peak concentrations of citrulline (Moinard et al. 2008; Cunniffe et al. 2016). Few studies investigating exercise performance have included concomitant measurements of peak plasma citrulline, which is likely due to the cost of such procedures. Indeed, Cunniffe et al. (2016) reported that following 12 g of CM plasma citrulline concentration was 343 ± 41 µM, compared to 39 ± 12 µM following the placebo. A similar magnitude of change was also observed for plasma ornithine concentration (9.5 ± 3.1 µM vs. 2.4 ± 1.6 µM). However, the time-course changes in these markers cannot be determined, as only a single blood sample was taken at 60 min. Equally, the 12-g dose ingested was higher than that typically used in CM research, therefore the changes in plasma citrulline concentration following the most common 8 g dose are unknown. Nonetheless, a study by Moinard et al. (2008) reported that following a range of doses of citrulline (2, 5, 10, and 15 g), peak concentration of citrulline occurred at approximately 1 h, however, this rapidly declined 15–30 min after the initial peak regardless of the dose ingested. Both plasma arginine and ornithine also displayed a similar pattern of peak and clearance. Further research investigating time-course changes in citrulline concentration following CM supplementation is required to determine the optimal strategy, although the best recommendation at the moment would be 1 h prior to exercise. It can also be concluded that the ‘ergogenic window’ is likely to be small and therefore determination of the peak absorption characteristics are likely to be important to securing an ergogenic benefit.

Most research investigating CM ingestion and exercise performance reports that a 2:1 ratio of citrulline:malate has been used. Recent research has challenged these reports, however, such that many CM manufacturers failed to reach the purported ratios (Chappel et al. 2018). Importantly, the ratios reported in a study using nuclear magnetic resonance spectroscopy reported most nutritional companies/suppliers only provided a CM ratio of approximately 1.6:1, with some as low as 1.1:1 (Chappel et al. 2018). Based on ingesting 8 g of CM with a 1.1:1 ratio, individuals are only ingesting 4.2 g instead of 5.3 g (the latter based on a 2:1 ratio). These potential quality control issues have ramifications for the existing body of work, future research, and its use by athletes in practice. Based on multiple studies using citrulline in isolation and reporting ergogenic effects (Gonzalez and Trexler 2020), it is likely the citrulline component of CM is vital to secure ergogenic benefits. It is plausible as a result that the equivocal findings in respect of CM ingestion and exercise performance could also be partly attributed to the amount of ingested citrulline being lower than intended. Researchers should therefore consider an independent assessment of supplement quality and authenticity to be able to gain assurances the dose ingested is as intended. If an independent assessment is not available, based on the findings from Chappel et al. (2018) only Trade Ingredients (www.tradeingredients.com) were close to the required ratio, and therefore this is the best-known source at this time.

The effects of citrulline supplementation on biomarkers associated with health outcomes have been previously investigated; however, limited evidence on CM exists. Indeed, Moinard et al. (2008) reported that ingestion of a range of doses between 2–15 g of citrulline had no adverse effect on hematological markers (leucocytes, polymorphonuclears, lymphocytes, monocytes, erythrocytes, Hb) or biochemical markers (calcium, total proteins, albumin, C-reactive protein, urea, creatinine, glucose, cholesterol, triacylglycerol). Unfortunately, no study as comprehensive as the Moinard et al. (2008) study exists for CM supplementation, although a small amount of research has studied health outcomes alongside supplementation (Brendahan et al. 2002; Casonatto et al. 2019). Indeed, Brendahan et al. (2002) supplemented CM chronically at 6 g^.^day^−1^ for 15 days, in a group of 18 sedentary males and reported no negative experiences. No objective health markers were measured, however, and the reports were subjective views from the authors. Casonatto et al. (2019), however, reported that following supplementation of 6 g of CM, both diastolic and systolic blood pressure was reduced over a 24-h period following exercise (40-min run/walk at 60–70% HR reserve) within a group of 40 hypertensive individuals. The applications of these findings would be difficult to apply to athletes, as they are likely to undertake more routine ingestion of CM compared to hypertensive patients. Considering the studies to date have only used an acute dose, and not used trained athletes, the safety of longer-term supplementation of CM requires further investigation. It is worth noting, however, that generally the ingestion of nitrate-based supplements are considered safe for consumption (Sindelar et al. 2012).

A common issue with ingestion of amino acids is the onset of gastrointestinal (GI) discomfort, whereby vomiting and diarrhea have been reported following ingestion of related amino acids such as arginine and ornithine in doses of between 6 and 12 g (Grimble 2007). Notably, the side effects reported with CM supplementation seem to be less severe. Indeed, Glenn et al. (2017) reported no differences in GI discomfort between CM supplementation and placebo following acute ingestion of 8 g within resistance-trained females, whilst larger doses of 12 g also seemed to be well-tolerated within a group of trained male cyclists (Cunniffe et al. 2016). These differences could be related to different mechanisms of citrulline uptake, such that it can be taken up from the lumen by multiple transport systems, including B^0,+^ (Bahri et al. 2008). In comparison, arginine and ornithine are rapidly saturated within the intestine and can therefore induce osmotic diarrhea. Moreover, these factors could explain why citrulline seems to have increased bioavailability compared to similar compounds, such as arginine (Breuillard et al. 2015; Moinard et al. 2008) and why larger doses can be tolerated. The findings for CM supplementation are therefore promising for real-world application in a performance setting, as no study to date has reported any significant GI discomfort following a range of doses.

## Effects of citrulline malate on exercise performance

Initial investigations have demonstrated that a single acute 8 g dose of CM ingested 1 h before exercise can enhance dynamic muscular endurance and strength performances, within resistance-trained males and females (Gonzalez and Trexler, 2020; Table 1). Perez-Guisado et al. (2010) used 41 resistance-trained males to perform four sets of barbell bench press at 80% 1RM (1-min rest between sets) following ingestion of 8 g CM, 1-h prior to exercise. Repetitions to failure in all but the first two sets of bench press before the workout were improved following CM ingestion. Performance responses have been replicated in resistance-trained females whilst performing six sets of bench press and plate-loaded leg press exercise at 80% 1RM (1-min rest between sets) using a similar dosing strategy (Glenn et al. 2017). Furthermore, in a series of investigations by Wax and colleagues (2015, 2016), ingestion of 8 g of CM 1-h before exercise increased the repetitions to failure during leg press and hack squat at 60% 1RM, and in a series of bodyweight exercises (chin-ups, push-ups), respectively. These changes were only subtle across these studies when expressed as an effect size (range: 0.23–0.59), which could suggest that CM’s ergogenic action might be more modest in such settings. Nonetheless, these small improvements across multiple sessions might improve training adaptation across a training block.

In contrast to the preliminary investigations assessing the ergogenic effects of CM supplementation on resistance exercise, no benefit to German Volume Training (GVT) protocols has been reported (Chappell et al. 2018a, 2018b). The ingestion of 8 g CM 1 h before exercise did not influence the total number of repetitions to failure during barbell curls (10 × 10 repetitions at 80% 1RM) (Chappell et al. 2018a) or isokinetic dynamometer leg curls (10 × 10 repetitions at 70% of concentric maximum force, 1-min rest between sets) (Chappell et al. 2018b). The latter protocol also showed no change in maximal isometric, concentric and eccentric force using this dosing strategy. Mitigating factors to explain the lack of performance improvement could be either the use of a CM compound disproportionate to the target ratio of citrulline and malate, or the reliability of the exercise protocol. Specifically, both studies by Chappell and colleagues (2018a, b) reported that the citrulline:malate ratio in the CM administered to participants was much lower than purported by the manufacturer (2:1 vs. 1.1:1). This equates to only around half of the stated dose of citrulline, which in turn, may have reduced the ergogenic potential. Lastly, GVT resistance training protocols do not seem to have any established test–retest reliability to date, which makes it difficult to determine small but meaningful changes in performance.

Some studies also confer no ergogenic effects of CM ingestion on dynamic or isokinetic muscular power indices (Farney et al. 2019; Gonzalez et al. 2018; Trexler et al. 2019a, 2019b). Indeed, Gonzalez et al. (2018) used a linear position transducer to examine the influence of 8 g CM (40 min before exercise) on power indices whilst performing 5 sets of up to 15 repetitions of barbell bench press exercise at 75% 1RM (2-min rest between sets). The authors reported no effect on peak power, mean power, fatigue index, or total repetitions performed by the group of resistance-trained males (*n* = 12). These findings agree with arguably the most comprehensive study to date by Trexler et al. (2019a) reporting ingestion of 8 g CM 2 h before exercise had no impact on peak torque, average torque, or total work during 5 sets of 30 maximal effort, concentric leg extensions (180°·s; 1-min rest between sets) compared to a placebo. The authors also reported no mechanistic evidence to suggest CM improves blood flow, metabolic efficiency, or lactate clearance. These findings have been corroborated during submaximal isotonic concentric leg extensions at 25% of maximal voluntary contraction torque (Trexler et al. 2019b). A caveat to both Gonzalez et al. (2018) and Trexler et al. (2019a; b) might be the ingestion timing of CM however, as both were outside of the 1-h before exercise timing used in studies reporting a positive effect on performance or where data suggests peak NO occurs (Moinard et al. 2008). Furthermore, it is unknown if the mechanisms of action would have been observed during full-body dynamic exercise where there is a higher aerobic and metabolic demand, as CM ingestion seems to be more appropriate for this type of exercise (*see proposed ergogenic mechanisms section*).

Positive effects of CM ingestion on cycling performance are scarce, with only three studies to date employing a randomised, placebo-controlled crossover study design (Glenn et al. 2017; Cunniffe et al. 2016; Gills et al. 2021). Indeed, Glenn et al. (2017) showed that 8 g of CM 1-h before exercise failed to improve mean power output or fatigue index during a cycling Wingate, within a group of 17 trained tennis players. Similarly, Cunniffe et al. (2016) observed no improvement during 10 × 15 s cycling sprints (30 s active recovery) despite the use of a higher 12 g dose of CM 1-h before exercise. The short-duration exercise protocols in these studies do not investigate the more aerobically derived mechanisms associated with CM supplementation, however, and this could explain the lack of ergogenic benefit. Nonetheless, in a contemporary study (Gills et al. 2021) employing a time to volitional tolerance (T_LIM_) protocol at a much lower exercise intensity (90% VO_2peak_), no improvement in cycling capacity was observed between ingestion of 8 g CM versus a placebo, within a group of 28 males (p = 0.94; PLA: 315.4 ± 137.7 s; CM: 314.1 ± 107.1 s). It is plausible that the lack of ergogenic effects could be attributed to the trained participant cohort in the cycling studies (VO_2peak_ range = 56.3 – 58.1 ml.kg^−1^.min^−1^), such that a recent point-counterpoint debate suggests due to an elevated NO at baseline compared to their untrained counterparts this could blunt the ergogenic effect (Hultström et al. 2015). Alternatively, the variation that is typically seen during T_LIM_ tests could explain the null effect, as these protocols require a large difference in performance to reach statistical significance compared to either fixed duration or distance time trials (Currell and Jeukendrup 2008). Considering these studies utilised the same dose and timing of ingestion as other studies demonstrating ergogenic effects in resistance exercise performance, the use of CM seems ineffective at improving cycling performance in trained individuals. Nonetheless, future research should combine CM ingestion and a time trial cycling bout with a predominantly aerobic demand to ascertain if a competitive edge could be gained.

Few studies have considered the ergogenic potential of CM supplementation in supporting the recovery of muscle function (da Silva et al. 2017) and/or adaptive properties of musculature in humans (Hwang et al. 2018a, b) (Table 1). Specifically, chronic supplementation of either 2 g of CM, 2 g L-citrulline, and 200 mg glutathione, or a placebo (2.52 g cellulose) per day over 8 weeks did not affect maximal muscular strength development (via bench press and leg press exercises) (Hwang et al. 2018a, b). Interestingly, whilst no significant difference was found, a large effect size (Hedges *g* = 1.8) was reported for reductions in fat mass at 8 weeks. A possible explanation for the null effect on performance and/or adaptation may be that the CM dose was too low, as this was significantly reduced compared to the dose typically used to produce ergogenic effects (2 g vs 8 g). However, this dose may be sufficient to help with a reduction in fat mass and therefore support overall athlete body composition. With this being the only study to date investigating chronic ingestion of CM, more research is required using higher doses that might subsequently improve the probability of securing any potential benefit to exercise performance and/or body composition.

A study investigating acute recovery from exercise has displayed null effects of CM supplementation (da Silva et al. 2017). The authors reported ingestion of 6 g of CM 1-h before exercise had no impact on the recovery of lower limb muscular endurance (one set at 100% of 10 RM leg press and hack squat), markers of muscle damage (creatine kinase), or electromyographic (EMG) activation compared to a placebo at 24, 48 and 72 h post-exercise. These author’s findings support earlier similar work displaying negligible changes in protein signaling and synthesis rates in elderly males, although this was with supplementation of L-citrulline (Churchward-Venne et al. 2014). It could be argued, however, that the exercise selected in da Silva et al. (2017) was not damaging enough to appropriately assess the recovery of muscle function. Specifically, between 24 and 72 h post-exercise the repetitions completed were similar that suggests the participants were not sufficiently fatigued at the 24-h time point. It is suggested a reduction of between 15–60% from baseline performance, which can persist for up to 2 weeks, is required to suggest exercise-induced muscle damage (Owens et al. 2019). In support, the creatine kinase concentrations were low (~ 350 UL^−1^), which compared to other literature, values greater than 1000 UL^−1^ are typically seen (Inman et al. 2018; Ehlers et al. 2002). Lastly, the crossover trial study design employed by da Silva et al. (2017) is arguably not the most appropriate to assess recovery from exercise-induced muscle damage; instead, a matched groups design could have been used to mitigate the repeated bout effect, particularly as da Silva et al. (2017) used recreational participants. As a result, based on the limited research to date and methodological limitations, further research is warranted.

## Conclusions and future directions

The lack of positive effects from CM supplementation within the existing literature is due to a number of factors, including the testing protocols not featuring a predominantly aerobic energy contribution, the lack of test–retest reliability of exercise protocols, dosing strategy (i.e., amount and timing), and the recent discovery of quality control issues with some manufacturers stated citrulline:malate ratios. Indeed, this diversity adds a level of additional noise to our ability to draw firm conclusions about the efficacy of CM supplementation on exercise performance or recovery from exhaustive exercise. Nevertheless, from the available evidence, an acute 8 g dose CM may, albeit not consistently, increase muscular endurance-strength performance (Table 1). This corroborates with a recent meta-analysis conducted at the time of writing this review, which also reported similar benefits (Vårvik et al. 2021). Whereas, there is little evidence to advocate its use in the production and maintenance of muscular power, maximal strength, recovery of muscular function, or supporting muscular adaptations currently (Table 1). Lastly, athletes wishing to explore NO enhancers are reminded that a good level of evidence exists for L-citrulline to improve exercise performance, and therefore may consider use of this supplement whilst the intricacies of CM supplementation are discovered (Gonzalez and Trexler 2020).

Future research should investigate the bioavailability of key variables, namely plasma NO, citrulline, and arginine following a range of doses of CM. Only one study exists that has reported this important data at the time of writing this review, and this did not include the malate component. Only at this point will the physiologically optimal dose of CM become clear. Due to the logistical and cost burden of conducting such a study, a simpler approach would be to assess various doses of CM (e.g., 8 vs. 10 vs. 12 g CM) on an exercise protocol that requires a predominantly aerobic energy demand and has high test–retest reliability. Furthermore, manufacturers are required to take more responsibility to guarantee that the ratio stated is what is contained within the product, and researchers/practitioners should be aware of this when sourcing their product for research and/or use with athletes. Finally, those who have the resources (primarily manufacturers but also researchers) should analyse the purity of the C:M ratio to ensure they have every opportunity of achieving an ergogenic effect.

## Abbreviations

CM: Citrulline malate
NO: Nitric oxide
ATP: Adenosine triphosphate
MAS: Malate aspartate shuttle
MDH: Malate dehydrogenase
NIRS: Near infrared spectroscopy
PFK: Phosphofructokinase
TCA: Tricarboxylic acid
HR: Heart rate
GI: Gastrointestinal
GVT: German volume training
VO_2peak_: Peak oxygen consumption
EMG: Electromyography
